# Trichoscopic Features of Eyebrow Trichotillomania: It Looks Similar to Scalp Trichotillomania

**DOI:** 10.5826/dpc.1002a40

**Published:** 2020-04-03

**Authors:** A. Tülin Güleç

**Affiliations:** 1 Department of Dermatology and Venereology, Başkent University Faculty of Medicine, Ankara, Turkey

**Keywords:** eyebrow, trichoscopy, trichotillomania, dermoscopy

## Introduction

Trichotillomania is a self-induced hair loss originating from pulling of one’s own hair repetitively in a compulsive manner, leading to significant hair loss. Although scalp hair is the most common site of involvement, it is also reported in other hair-bearing areas of the body such as eyelashes, eyebrows, and beard. Trichoscopic criteria have been well described for scalp trichotillomania including mainly broken hairs at different lengths, short hairs with trichoptilosis, coiled hairs, hook hairs, tulip hairs, flame hairs, and V-sign [1]. Yet, to our knowledge there are no data regarding the trichoscopic features of this alopecia on eyebrows.

## Case Presentation

**Figure 1:**
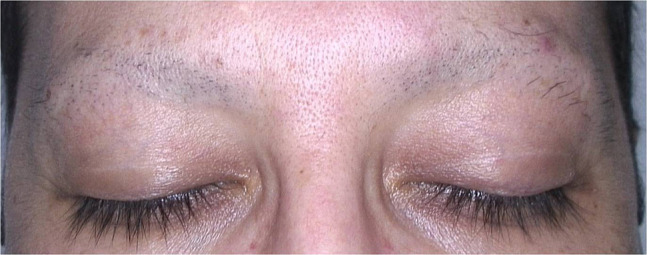
Eyebrow trichotillomania. Almost complete loss of eyebrows with a few hairs on the lateral sides, and black dots with some broken hairs on the remaining main part.

**Figure 2:**
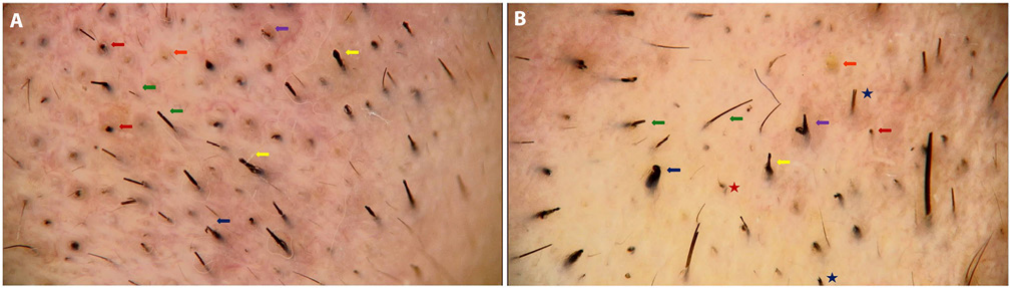
Trichoscopic features of eyebrow trichotillomania. (A) Black dots (red arrows), broken hairs at different lengths (green arrows), yellow dot (orange arrow), hook hair (blue arrow), tulip hairs (yellow arrow), V-sign (purple arrow). (B) Broken hairs at different lengths (green arrows), hook hair (blue arrow), flame hair (red star), tulip hair (yellow arrow), V-sign (purple arrow), yellow dot (orange arrow), short hairs with trichoptilosis (blue stars), black dot (red arrow) (original magnifications: A,B, ×20).

A 40-year-old man presented with a years-long history of numerous nevi. While he was being examined by a computerized polarized light videodermatoscope (FotoFinder Dermoscope; TeachScreen Software GmbH, Bad Birnbach, Germany), almost complete loss of his eyebrows was noticed. Dermatological examination showed a few hairs on the lateral sides, while there were just black dots and some broken hairs on the remaining part of the eyebrows (Figure 1). Trichoscopic examination revealed various findings, namely broken hairs at different lengths, coiled hairs, hook hairs, short hairs with trichoptilosis, V-sign, flame hairs, tulip hairs, black dots, and a few yellow dots (Figure 2, A and B). After we took a careful history, he reported that he had been pulling out his eyebrow hairs during stressful times for the past 4 years.

## Conclusions

The main clinical differential diagnosis of trichotillomania is patchy alopecia areata. However, it creates a diagnostic challenge in particular when it occurs on a body site other than the scalp, and/or if the patient denies the habit of hair pulling. Besides, eyebrow alopecia may be secondary to numerous causes such as hypothyroidism, chemotherapy, secondary syphilis, leprosy, and frontal fibrosing alopecia in addition to alopecia areata [2]. Furthermore, trichoscopic findings are not necessarily identical in alopecias that can involve both scalp and eyebrow hairs, as in frontal fibrosing alopecia. It presents red or gray dots on the eyebrow area, while loss of follicular openings, perifollicular erythema, and scaling are the main features on the frontal scalp. Yet, in the present case, we could easily diagnose trichotillomania when we observed identical trichoscopic features of scalp trichotillomania and confirmed it by the appropriate history. Consequently, we suggest that trichoscopy is also beneficial for the clinical diagnosis of eyebrow trichotillomania.
